# What a paediatrician should know about congenital clubfoot

**DOI:** 10.1186/s13052-020-00842-3

**Published:** 2020-06-04

**Authors:** Daniela Dibello, Valentina Di Carlo, Giulia Colin, Egidio Barbi, Anna M. C. Galimberti

**Affiliations:** 1 Institute for Maternal and Child Health IRCCS Burlo Garofolo, Via dell’Istria 65/1, Trieste, Italy; 2 University of Trieste, Piazzale Europa 1, Trieste, Italy

**Keywords:** Clubfoot, Talipes, Equinovarus, Management, Pediatric

## Abstract

Clubfoot is the most frequent congenital malformation of the foot, affecting more than 1–2 subjects per 1.000 newborns. Without appropriate treatment, a child with congenital clubfoot will never be able to walk physiologically with a dramatic impact on the quality of life. In the last decades, different corrective solutions have been proposed, and there is rising scientific evidence that the Ponseti non-invasive method is safe and effective in the treatment of the clubfoot. So, what should a general paediatrician know about this condition and what should he concretely do in the suspect of a congenital clubfoot?

## Background

The talipes equinovarus congenital foot, also known as congenital clubfoot, is the most common congenital malformation of the foot. One or two, per 1000 newborns are affected [1], with a male to female ratio of 1 to 2. This condition is particularly frequent in Developing Countries (80% of overall cases). In 50% of cases, it affects both feet [2]. Without adequate treatment children with congenital clubfoot will not walk physiologically and will not be able to live a normal life. Paediatricians have a critical role in the early detection of this condition, starting from the first evaluations of the newborn. The prompt referral to the specialist is crucial for these children to obtain a plantigrade and functional foot. This article aims to provide the general paediatrician with essential knowledges for proper clubfoot management. The scientific literature supports the efficacy of Ponseti method for this pathology, and we tried to simplify and explain the therapeutic process and the general management.

### What is clubfoot?

**Fig. 1:**
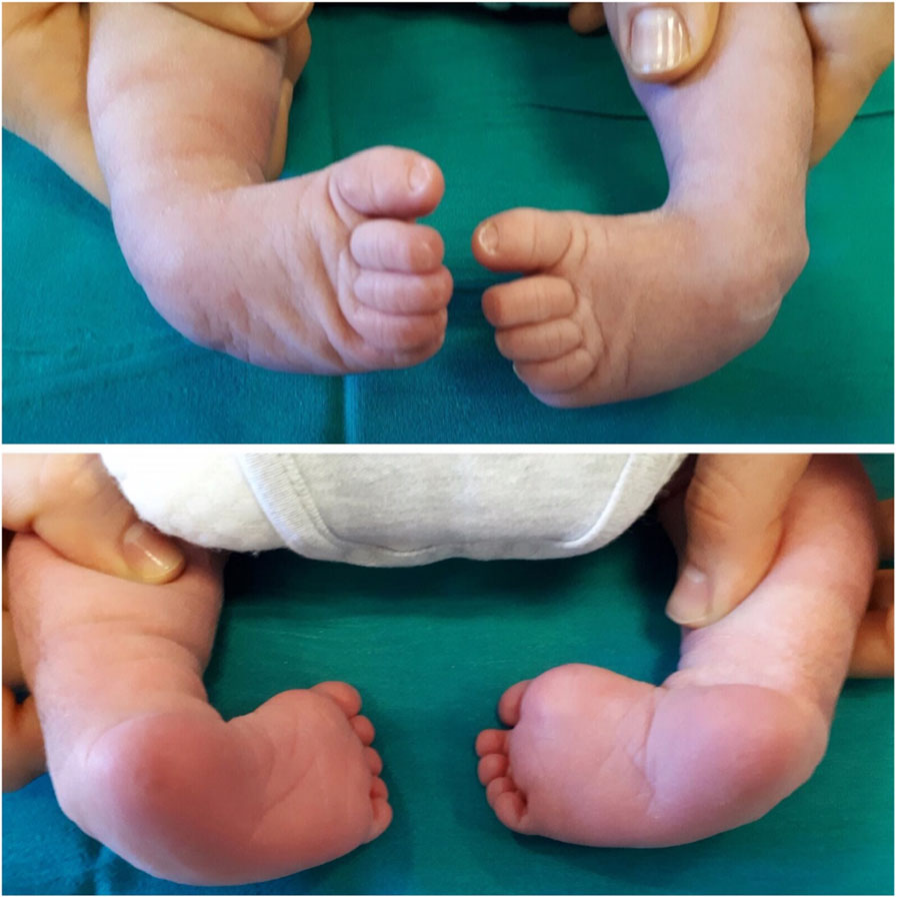
Frontal and posterior view of congenital idiopathic clubfoot

**Fig. 2:**
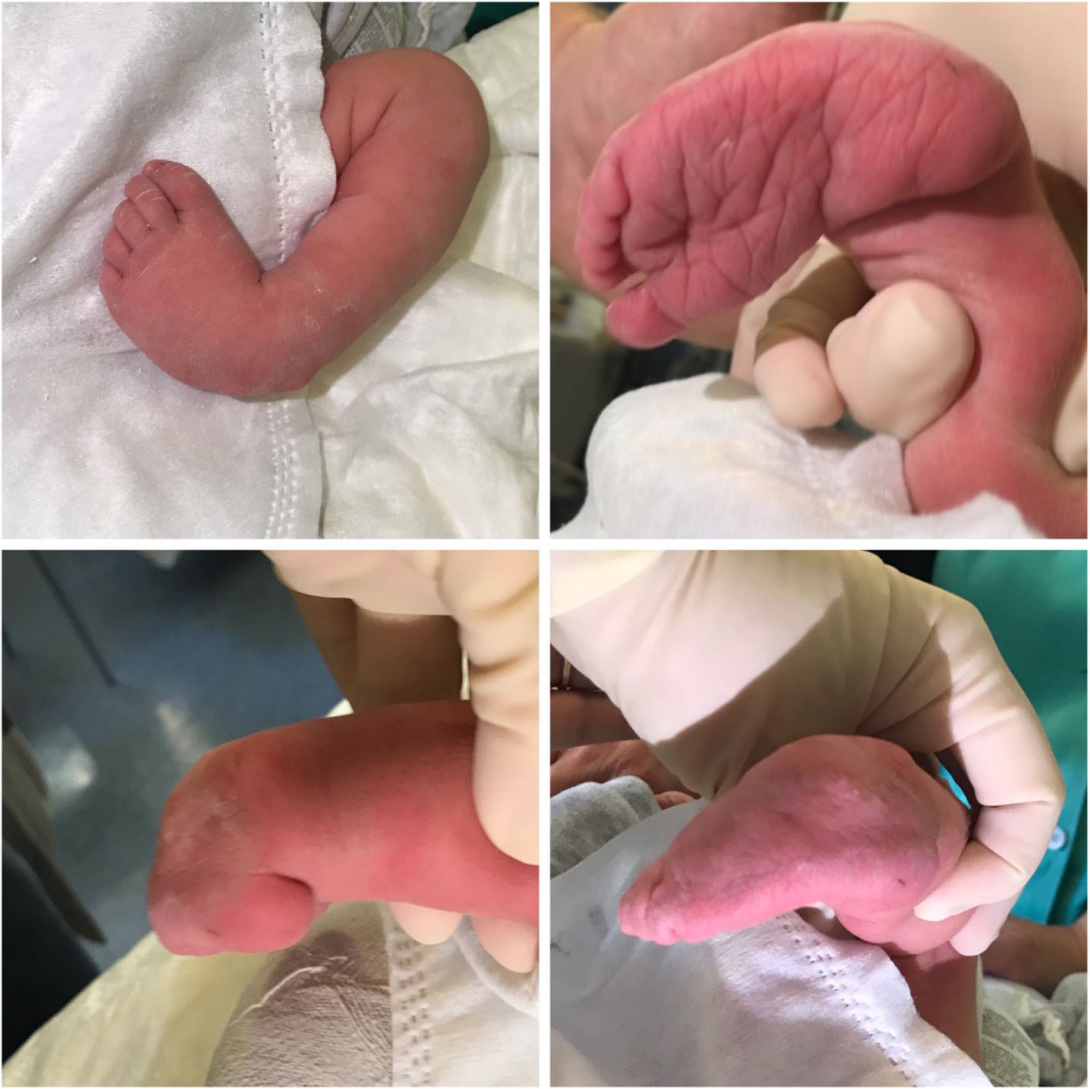
Severe clubfoot. legend: severe clubfoot (Pirani 6, Manes-Costa 3)

Congenital clubfoot is a malformation characterized by a torsion of the longitudinal axis of the foot, secondary to a malalignment of the calcaneo-talar-navicular complex. The foot’s sole is rotated medially and this leads the child to walk on the foot sides (Fig. 1). We can detect four different anomalies; **CAVE** is a mnemonic tip to remember them all (Fig. 2):

1. *Midfoot****cavus****deformity* (the sole of the foot “looks” upwards).
2. *Metatarsus****adductus*** (the fingers point inside with concavity of the medial foot margin).
3. *Hindfoot****varus****deformity* (medial deviation of the longitudinal axis of the calcaneus).
4. *Hindfoot****equinus****deformity* (extreme plantar flexion).

There are two main types of congenital clubfoot: idiopathic (80% of cases) and secondary clubfoot (20% of cases). The idiopathic congenital clubfoot is a multifactorial condition that includes environmental, vascular, positional, and genetic factors. Clubfoot has a tendency to segregate in families: the risk of developing congenital clubfoot is 25% when a first-degree relative is affected. Several studies and observations suggest the existence of different genes and inheritance patterns involved [3]. On the other hand, up to 20% of cases of congenital clubfoot is associated to other anomalies (e.g., myelomeningocele) and could be secondary to a specific genetic condition such as *Moebius syndrome, neurofibromatosis* and *multiple congenital arthrogryposes* [4]. The congenital clubfoot could also show more complex anatomic features that typically present a shorter and more rigid foot, in which there is a marked curvature of the midfoot (metatarsal equinism) with deep skin folds. In these cases we talk about an “atypical clubfoot” [5]. Clinical features of clubfoot may already emerge in prenatal diagnostics, but its ultrasonographic diagnosis appears more likely between the 18th and the 24th week of pregnancy [6].

### How to perform a focused examination?

**Fig. 3:**
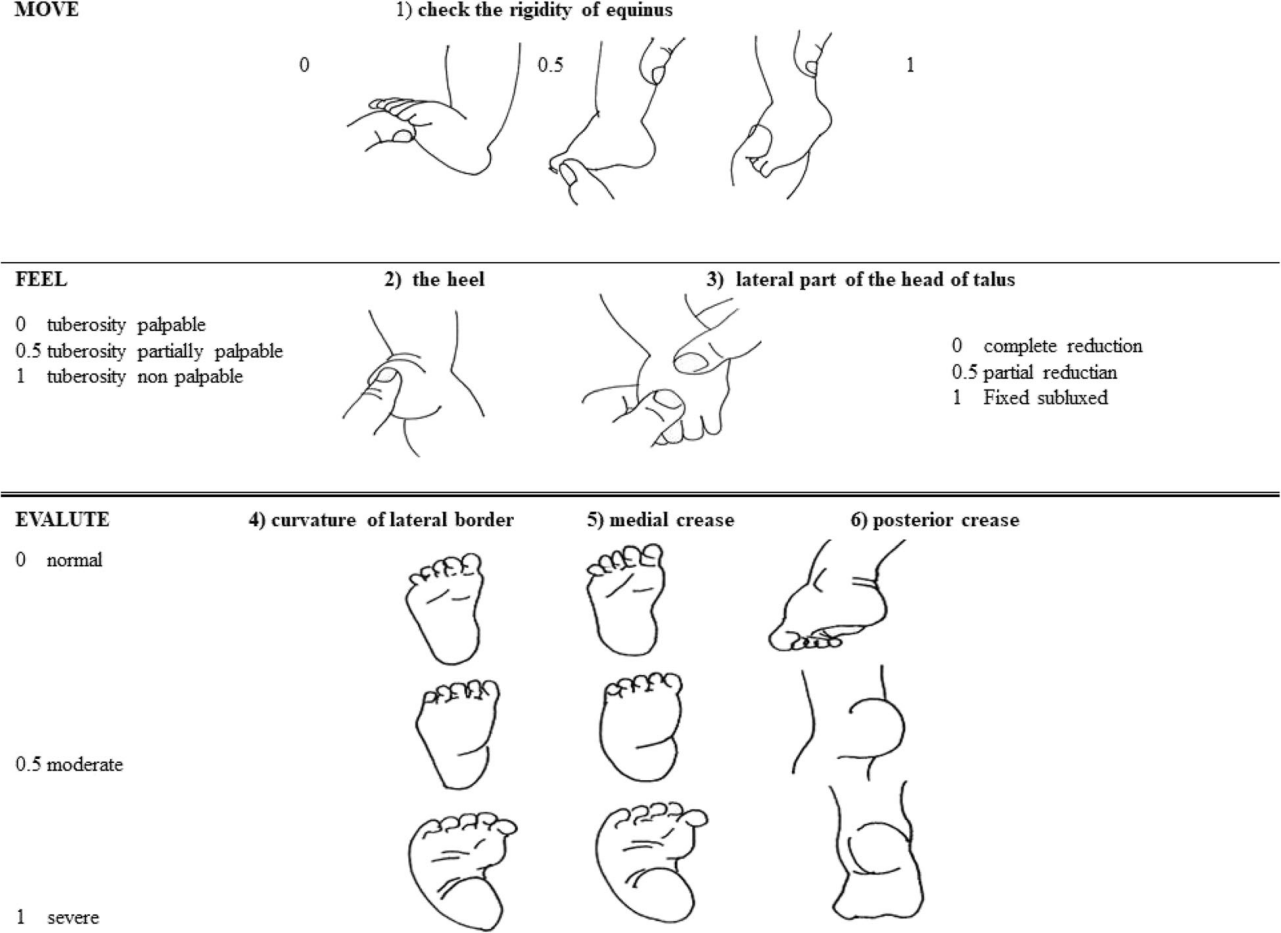
Pirani clubfoot- score. legend: this is a 6-item scale, in which every point has a 0 to 1 score (0 if normal, 0.5 if moderate-mild deformity is present, 1 with a severe malformation); the higher the score is, the worst is the deformity

The foot needs to be evaluated in their complexity: general morphology, presence of skin folds, muscular and tendon malleability and flexibility of the deformation. Flexibility is the most relevant element that influences the prognosis: the more malleable and easy to move the foot, the better the prognosis. There are several classification systems of the clubfoot, for example Manes-Costa’s classification, Pirani’s score (Fig. 3) or Dimeglio classification. These scores are valid prognostic tools and can be used in the follow-up process. A high score at presentation may indicate that a longer and more complex treatment will be required. Whenever a congenital clubfoot is detected, a complete examination is mandatory to rule out other neuro-musculoskeletal problems, such as signs of occult spinal dysraphism, developmental dysplasia of the hip (DDH) or congenital torticollis.

It is essential to differentiate clubfoot from two other disorders: postural equino-varus foot and metatarsus adductus. The postural equino-varus is a functional malposition of the foot caused by the deformed position that the fetus used to hold in the maternal womb. In this case, there may be some degrees of forefoot adduction, but it will be flexible at the manipulation, and there will be no Achilles tendon contracture. Generally, the postural equino-varus resolves spontaneously; nevertheless, it is good practice to monitor the progression (in premature newborn, a clubfoot can sometimes hide behind a postural equino-varus) [7]. The adductus metatarsus is another common deformity characterized by an adducted forefoot with a curvature of the lateral edge of the foot, but without the other features of clubfoot. Also, this disorder is secondary to the intrauterine postural modeling of the feet and generally resolves spontaneously (casting may be necessary if the deformity is rigid).

### Which therapy?

**Fig. 4:**
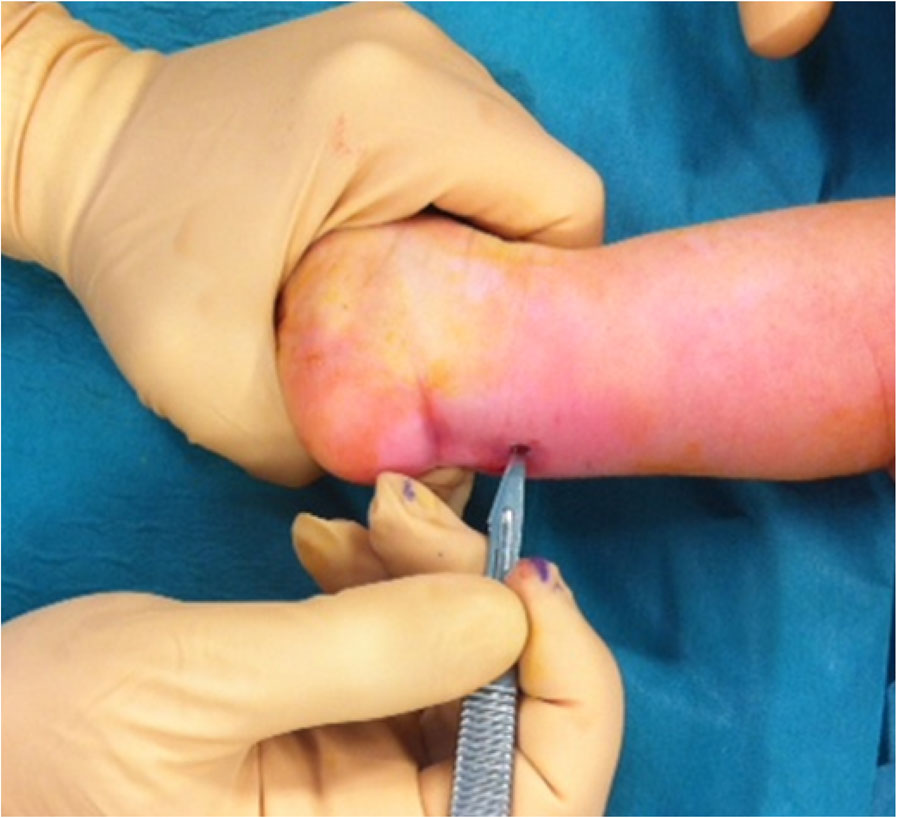
Percutaneous tenotomy of the Achille’s tendon

Over the decades the surgical treatment of congenital clubfoot has mostly been abandoned, as it was associated with complications, with the final result of a foot that would hardly reached full functionality, due to retractions and scars, secondary to the surgery itself. Although some complex and atypical clubfeet still need surgical treatments at first [7], the available evidence has definitively confirmed the effectiveness of the Ponseti non-invasive method [1, 8, 9]. It consists of gentle manipulations of the foot followed by the application of plaster casts, which are kept in place for 5-7 days, during which muscles and ligaments adapt to the new position. At the end of the 5-7 days period, the cast is removed, the foot (which would become softer and more prone to be moulded) is revaluated and progressively manipulated to maintain, through the application of a new cast, a new position [2, 8]. The procedure is repeated until normal foot alignment is achieved (on average, about five to six plaster casts are required). Percutaneous Achille’s tenotomy (Fig. 4) could be required if equinus deformity persists at the end of the casting phase. The procedure takes about 5 min and consists of a millimetric posterior skin incision through which the tendon section is achieved. After the tenotomy, plaster is applied to allow the tendon to heal in elongation for about 20 days. In order to maintain the correct position of the foot,it is necessary to wear, an orthopedic brace until 5 years of age. The brace must be worn for 23 h a day for the first 3 months. Over time, the child may gradually decrease the use of the brace during the day:: after the first months, it is necessary to wear the cast for at least 18–19 h with a gradual reduction in the use of 1 h per month up to a maximum of 12 h without the brace. When the patient begins to walk independently, the brace is usually only held overnight until the age of five.. Compliance with the splinting programme is crucial to prevent recurrences [10], and the general paediatrician has a critical role in supporting the family during the brace-phase.

To conclude, the treatment of an “atypical clubfoot” is more challenging and difficult and often requires a higher number of plaster casts.

### What management should a paediatrician provide?

Radiological diagnostic evaluations, such as foot X-rays or ultrasounds, are usually not necessary in addition to the clinical assessment. In the case of very complex clubfoot, it could be reasonable to evaluate the possibility of an underlying process or other associated malformation. A co-existent hip dysplasia, myogenic torticollis, or other orthopedic conditions should always be ruled out. The paediatrician also has to evaluate the child to exclude a systemic condition (such as a neuromuscular disease or a syndromic illness – see Table 1). Paediatricians are also in charge of preparing the family for the therapeutic process. Reassurance and providing a direct link to an experienced centre are the first steps. Operational timeliness is essential in terms of outcomes so the newborn needs to be referred to the specialist as soon as possible, e.g., in the very first days of life. The relapses of the clubfoot are not uncommon (about 5–10%), either with conservative and surgical methods. Consequently, paediatricians should play a relevant role in supporting the family and monitoring compliance with the constant use of the orthopedic brace, which represents the main factor in preventing the recurrence of malformation.

**Table 1:** from B. Sadler, C. A. Gurnett, and M. B. Dobbs “The genetics of isolated and syndromic clubfoot”, Journal of Children Orthopaedics Jun 2019

Condition/syndrome name	Known genes
Autosomal Dominant Larsen Syndrome, Recessive spondylocarpotarsal syndrome	FLNB
Barth Syndrome	TAZ
Bruck Syndrome	PLOD2, FKBP10
Carey-Fineman-Ziter Syndrome	MYMK
Catel-Manzke Syndrome	TGDS
Charcot-Marie-Tooth Disease Type 4D	NDRG1
Diastrophic dysplasia	SLC26A2
Ehlers-Danlos Syndrome, Musculocontractural type 1	CHST14
Ehlers-Danlos Syndrome, Musculocontractural type 2	DSE
Ehlers-Danlos Syndrome, vascular type	COL3A1
Epileptic Encephalopathy	AARS
Joubert Syndrome	ATXN10, TCTN2
Loeys-Dietz Syndrome	TGFBR1, TGFBR2, SMAD3, TGFB2, TGFB3
Marfan Syndrome	FBN1, TGFBR, TGFBR1, TGFBR2, SMAD3, TGFB2, SKI
Moebius Syndrome	PLXND1, REV3L
Multiple Epiphyseal Dysplasia	COL9A1, COL9A2, COL9A3, COMP, MATN3, SLC26A2
Multiple Synostosis Syndrome	GDF5
Peroxisome biogenesis disorder 7A	PEX26
Recessive axonal Charcot-Marie-Tooth Disease	LMNA, GDAP1
Recessive Larsen Syndrome, Humero-Spinal Dysostosis, Spondyloepiphyseal dysplasia	CHST3
Richieri-Costa – Pereira Syndrome	EIF4A3
Santos Syndrome	WNT7A
Saul-Wilson Syndrome	COG4
Schpritzen-Goldberg Syndrome	SKI
TARP Syndrome	RBM20
Van Maldergem Syndrome 2	DCHS1, FAT4

### Four golden rules for an adequate approach

1. The earlier, the better: if you detect a clubfoot contact a local orthopedic surgeon who can take care of the referral to a clubfoot center, preferably within 48 h but not more than 1 week after the delivery [7];
2. Motivate the parents: to improve the compliance with the use of the brace to minimize the recurrences [7];
3. In case of a severe clubfoot: inform the parents of the increased risk recurrences;
4. Clubfoot is diagnosed through clinical evaluation, and usually, a radiological evaluation is not necessary. The orthopedic surgeon can choose to perform a radiological evaluation in selected cases (e.g., in front of a weak response to treatment or severe relapses) [7].

## Conclusion

Clubfoot is a highly invalidating condition all over the world; the absence of an adequate treatment will lead to dramatic consequences on the quality of life of the patients, with a high social burden. The rapid recognition of deformity and immediate reference to the orthopedic specialist are the key elements for effective treatments.

## Data Availability

Not applicable.
